# Lemierre’s syndrome: A rare complication of acute bacterial pharyngitis

**DOI:** 10.4102/sajid.v39i1.606

**Published:** 2024-04-09

**Authors:** Pierre Joubert, Muhammed S. Moosa

**Affiliations:** 1 Department of Internal Medicine, Faculty of Health Sciences, New Somerset Hospital, University of Cape Town, Cape Town, South Africa; 2 Department of Internal Medicine, Faculty of Health Sciences, Groote Schuur Hospital, Cape Town, South Africa

**Keywords:** Lemierre’s syndrome, sepsis, thrombosis, septic emboli, strep pyogenes, streptococcus, neck mass, jugular thrombophlebitis

## Abstract

Lemierre’s syndrome is a rare clinical syndrome of septic thrombophlebitis following a bacterial oropharyngeal infection. Lemierre’s syndrome can be difficult to recognise and has significant morbidity. We report the case of a young man with Lemierre’s syndrome caused by *Streptococcus pyogenes*, who responded well to 2 weeks of beta-lactam therapy.

**Contributions:**

This case report summarises the key presenting features of Lemierre’s syndrome and provides a brief literature review considering the South African context.

## Introduction

Lemierre’s syndrome (LS), also known as post-anginal sepsis or necrobacillosis, is a rare clinical syndrome first described in 1936 by Andre Lemierre. He described the syndrome in healthy patients who developed septic thrombophlebitis of the internal jugular vein (IJV) following a bacterial oropharyngeal infection.^1,2^ The incidence of LS is reported to be 1 in 1 000 000 with majority (90%) patients being between ages 10 years and 30 years.^3^ However, a retrospective study from Denmark reported 9.4 in 1 000 000 cases per year in young adults.^4^ The most common causative bacterium is *Fusobacterium necrophorum*.^5^ However, other bacteria including streptococcal species, *Staphylococcus aureus*, bacteroides, lactobacillus, peptostreptococcus and *Eikenella corrodens*, have been reported.^2,6,7^ Mortality from LS has declined from 90% in the pre-antibiotic era to 4% – 10% currently but still causes significant morbidity because of its elusive nature and increased antibiotic resistance.^2,6,7^

In sub-Saharan Africa, upper respiratory tract infections are common. However, only a few cases of LS have been reported.^6,8^ We report a young man who presented to a regional hospital in Cape Town with suspected LS.

## Presentation

A previously healthy 20-year-old male, presented to the emergency department of a regional hospital in Cape Town with a history of acute pharyngitis 3 weeks ago while camping. A week later, he noticed painful and swollen knees causing difficulty in weight bearing, as well as crusting skin lesions on his lower limbs. Two weeks later, he developed a fever, loss of appetite and a progressively enlarging, painful right-sided neck mass. He had neither a history of medical conditions nor substance abuse (including intravenous drug abuse).

On examination, he had an antalgic gait, a temperature of 38 °C and a palpable large tender mass lateral to the right sternocleidomastoid muscle. He had bilateral tender, swollen knees with multiple small eschar-like lesions on both lower limbs. He had no signs of conjunctivitis, urethritis, rheumatic fever nor stigmata of infective endocarditis. Our two leading differential diagnoses at this stage included: (1) a neck abscess with septic or reactive arthritis and (2) rickettsia infection.

**FIGURE 1:**
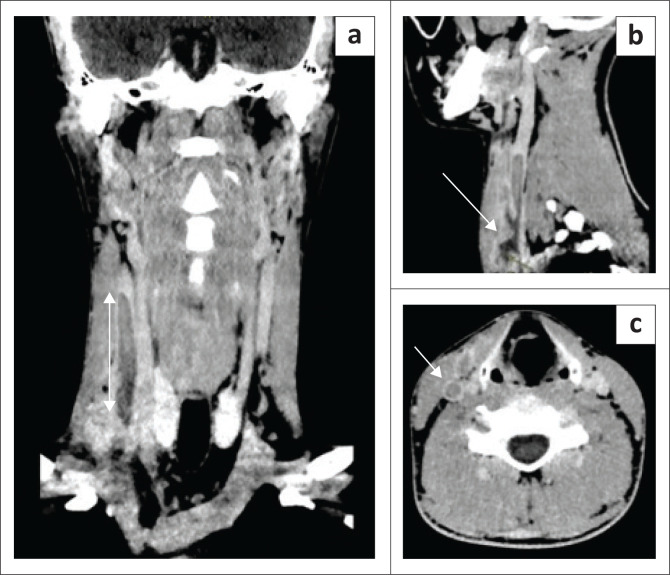
(a) Coronal contrasted computer tomography displaying right internal jugular vein thrombosis. (b) Sagittal computer tomography displaying thrombosis as well as soft tissue extension into clavicular bulk of SCM. (c) Cross-section view of septic thrombus in the internal jugular vein.

On further investigation, his urine dipstick, chest radiograph and renal function were normal. He had elevated serum liver enzymes: with alanine aminotransferase of 213 U/L, aspartate aminotransferase 200 U/L, alkaline phosphatase 280 U/L and gamma glutaryl transferase 204 U/L. The C-reactive protein was 287 mg/L. Rickettsia Immunoglobulin G and Immunoglobulin M, leptospira IgM, viral hepatitis A IgM, viral hepatitis B surface antigen and viral hepatitis C antibody were all negative. Anti-streptolysin O titre and Anti-DNase B were 2900 IU/mL and 1210 IU/mL, respectively, suggesting recent streptococcal infection. *Streptococcus pyogenes* was identified on blood culture with susceptibility to penicillin and ampicillin. Synovial fluid aspirate from the right knee showed a polymorphonuclear cell predominance, but no bacterial growth was observed on culture. Computer tomography (CT) of the neck (Figure 1) demonstrated a right internal jugular thrombus in addition to a small low-density collection in the right sternocleidomastoid muscle with no deep neck space infection demonstrated. A CT abdomen and thrombophilia screen were unremarkable.

Our patient was treated with intravenous ceftriaxone, warfarin and physiotherapy with resolution of his swollen joints and skin lesions. His lower limb skin lesions were suspected to be impetigo or septic emboli. The swollen knees were attributed to a possible treated septic arthritis or reactive arthritis. Septic emboli to the large joints have been described in LS.^9^ He was discharged home 14 days after admission on long-term anticoagulation. The patient underwent follow-up CT at 6 months showing complete resolution of IJV thrombus and warfarin was stopped.

## Discussion

The pathophysiology of LS is poorly understood, but it is postulated that bacteria invade the pharyngeal mucosa and spread to the peritonsillar and IJVs resulting in septic thrombosis.^3,7^ Patients with LS may present with pharyngitis, tonsillitis and a tender mass over the angle of the mandible because of cervical vein thrombosis referred to as ‘Cord’s sign’.^7^ A diagnosis of LS is usually based on a triad of recent oropharyngeal infection, thrombosis of the IJV and complications of septic emboli.^10^ Septic pulmonary emboli (PE) were noted in 37.6% of patients with LS in a recent metanalysis.^2^ Less common complications of embolism include septic arthritis, osteomyelitis, pericarditis, brain emboli and meningitis.^2,3,7^

The management of LS is centred around early initiation of broad-spectrum antibiotics. Current guidelines recommend an initial combination of penicillin and metronidazole^6^ There is no consensus on the use of anticoagulation in patients with LS because of a lack of randomised control trials.^5^ A recent metanalysis showed no significant relationship between anticoagulation and recanalisation.^2^ However, there have been anecdotal and non-observational studies suggesting that anti-coagulation hastens clinical recovery.^5^ Anticoagulation is specifically recommended in patients where adequate antimicrobial therapy has been delayed, where bacteraemia persists, an underlying thrombophilia is present and where intracranial thrombosis is identified.^11^ Surgical intervention is reserved in cases where the drainage of abscesses or collections are warranted.^11^

Despite a high burden of infectious diseases in South Africa, only two other cases of LS have been reported in the literature.^7,8^ The first case report describes a 20-year-old man who presented with left-sided facial swelling and a lower respiratory tract infection because of *Escherichia coli*.^8^ An oropharyngeal abscess with secondary IJV thrombophlebitis was diagnosed, and it was presumed that this was the source of septic PE. The patient was treated with warfarin and combination antibiotics, cefuroxime, gentamicin and metronidazole and showed radiological improvement on routine follow-up.^8^

The second case was that of a 14-year-old boy with chronic otitis media, who developed severe sepsis and meningitis. This patient had spread of the septic thrombus from IJV into the sigmoid sinus and extending to the right transverse dural sinus. Blood cultures were negative while *Proteus vulgaris* and *Pseudomonas aeruginosa* were cultured from ear swabs. Despite being treated with gentamicin, ertapenem and vancomycin, the patient eventually demised from septic shock.^6^

Currently, there are no reported case series assessing antimicrobial resistance in patients with LS. There are isolated case reports of resistant *Klebsiella pneumonia* causing LS, and phagocytic resistance has been suggested as the mechanism of resistance of certain capsular serotypes.^12^ While *S. pyogenes* is considered universally sensitive to penicillin and ampicillin because of lack of beta-lactamase production, regular surveillance of antimicrobial resistance in LS should be conducted.^13^

## Conclusion

The complications of LS can vary from mild to fatal. Therefore, delays in treatment with antibiotics should be avoided. Further research is needed to identify possible risk factors that may be involved in the pathogenesis of this disease, because most untreated upper respiratory tract infections do not result in this potentially lethal complication. It is helpful to consider LS as part of a differential diagnosis in the setting of a recent oropharyngeal infection, a neck mass or signs of septic emboli. This should be done for especially those patients presenting with symptoms of septic PE. Knowledge of the stages of disease progression in LS, as well as initial management, may be valuable for primary healthcare providers.

## References

[1] Lemierre A. On certain septicaemias due to anaerobic organisms. Lancet. 1936;227(5874):701–703. 10.1016/S0140-6736(00)57035-4

[2] Gore MR. Lemierre syndrome: A Meta-analysis. Int Arch Otorhinolaryngol. 2020;24(3):379–385. 10.1055/s-0039-3402433 PMC7394644 32754251

[3] Allen BW, Anjum F, Bentley TP. Lemierre syndrome [homepage on the Internet]. Treasure Island, FL: StatPearls Publishing; 2023 [cited 2023 Jun 10]. Available from: https://www.ncbi.nlm.nih.gov/books/NBK499846/29763021

[4] Bank S, Jensen A, Nielsen HM, Kristensen LH, Voldstedlund M, Prag J. *Fusobacterium necrophorum* findings in Denmark from 2010 to 2014 using data from the Danish microbiology database. J Pathol Microbiol Immunol. 2016;124(12):1087–1092. 10.1111/apm.12606 27704629

[5] Lee W Sen, Jean SS, Chen FL, Hsieh SM, Hsueh PR. Lemierre’s syndrome: A forgotten and re-emerging infection. J Microbiol Immunol Infect. 2020;53(4):513–517. 10.1016/j.jmii.2020.03.027 32303484

[6] Roos M, Harris T, Seedat R. Fatal lemierre’s syndrome as a complication of chronic otitis media with cholesteatoma. S Afr J Child Health. 2016;10(4):231–232. 10.7196/SAJCH.2016.v10i4.1074

[7] Amarnani S, Ranjan A. Lemierre’s syndrome: A lethal complication of acute tonsillitis. Cureus. 2022;14(10):8–13. 10.7759/cureus.30072 PMC9639786 36381870

[8] Such R, Joseph E. Lemierre’s syndrome – The uncommon cold. S Afr J Radiol. 2005;9(2):22. 10.4102/sajr.v9i2.87

[9] Olivier JB, Al-Hourani K, Bolland B. Lemierre’s syndrome; a rare cause of septic arthritis. BMJ Case Rep. 2017;2017:1–4. 10.1136/bcr-2017-220110 PMC5614224 28500126

[10] Moher D, Liberati A, Tetzlaff J, et al. Lemierre’s syndrome: Current perspectives on diagnosis and management. Infect Drug Resist. 2016;9(7):221–227. 27695351 10.2147/IDR.S95050 PMC5028102

[11] Chaker K, Berrada O, Lyoubi M, et al. Lemierre’s syndrome or re-emerging disease: Case report and literature review. Int J Surg Case Rep. 2021;78:151–154. 10.1016/j.ijscr.2020.12.015 33352443 PMC7753231

[12] Hwang SY, Shin SJ, Yoon HE. Lemierre’s syndrome caused by *Klebsiella pneumoniae*: A case report. World J Nephrol. 2021;10(5):101–108. 10.5527/wjn.v10.i5.101 34631480 PMC8477271

[13] Kebede D, Admas A, Mekonnen D. Prevalence and antibiotics susceptibility profiles of *Streptococcus pyogenes* among pediatric patients with acute pharyngitis at Felege Hiwot Comprehensive Specialized Hospital, Northwest Ethiopia. BMC Microbiol. 2021;21(1):1–10. 10.1186/s12866-021-02196-0 33941090 PMC8091706

